# Path Analysis Associations Between Perceived Social Support, Stressful Life Events and Other Psychosocial Risk Factors During Pregnancy and Preterm Delivery

**DOI:** 10.5812/ircmj.11271

**Published:** 2013-06-05

**Authors:** Arash Mirabzadeh, Mahrokh Dolatian, Ameneh Setare Forouzan, Homeira Sajjadi, Hamid Alavi Majd, Zohreh Mahmoodi

**Affiliations:** 1 Department of Social Determinants of Health Research Center, University of Social Welfare and Rehabilitation Sciences, Tehran, IR Iran; 2 Department of Psychiatric, University of Social Welfare and Rehabilitation Sciences, Tehran, IR Iran; 3 Department of Social Determinants of Health Research Center, University of Social Welfare and Rehabilitation Sciences, Tehran, IR Iran; 4 Department of Biostatistics, School of Paramedical Sciences, ShahidBeheshti University of Medical Sciences, Tehran, IR Iran; 5 Faculty of Nursing and Midwifery, Department of Midwifery, Alborz University of Medical Sciences, Karaj, IR Iran

**Keywords:** Social Support, Depression, Anxiety, Stressful Life Events, Path Analysis, Stress

## Abstract

**Background:**

Although several socio-medical risk factors have been identified for preterm labor, there is a gap in understanding the underlying etiology of preterm labor.

**Objectives:**

The current study aimed to analyze the relationship pathway of perceived social support, stressful life events, and other psychosocial risk factors during pregnancy with incidence of preterm labor.

**Materials and Methods:**

In a prospective cohort study in four hospitals in Tehran, 500 pregnant women in their 24th to 28th gestational weeks were studied. They filled out a self-report questionnaire on perceived social support, depression, anxiety, stress and stressful life events. Sociodemographic characteristics were also assessed. The participants were followed up until labor, and the data about mother and the newborn were collected after labor. The data were analyzed by SPSS 16 and Lisrel 8.8 software programs using pathway analysis.

**Results:**

The final path model fit well (CFI = 0.96; RMSEA = .064). The results showed that depression, anxiety, and stress (β = -0.18) directly, and stressful life events indirectly (β= -0.0396) had the most predict on gestational age at labor. Perceived social support, directly through socioeconomic status (β=0.25), and indirectly through stress, depression and anxiety (β= -0.26) affected the gestational age at birth (β= 0.0468).

**Conclusions:**

The current study showed that supporting pregnant mother moderates psychological problems such as stress, anxiety, and depression, and hence reduces preterm labor.

## 1. Background

Despite advances of medical sciences in the diagnosis and treatment of diseases, preterm birth remains a global problem in developed and developing countries (1, 2). Preterm birth means termination of pregnancy before 37 weeks of gestational age, and it is one of the main causes of infant mortality, claiming over one million infants’ lives each year(3-8). Birth is an important and critical stage in human life, and normally in the process of labor, human is faced with many physical and psychological stresses. Given the lack of necessary development of body systems, preterm labor and birth of a premature baby make this process much harder and more complex (9). There is no published global statistics on the incidence of preterm birth. The overall prevalence of preterm birth is 9.6%, but distribution of these births is not uniform worldwide, and 85% of these cases occur in Asia and Africa (10). Its prevalence has been reported as 5% in developed and 25% in developing countries (5, 8). In Iran, the prevalence has been reported between 5.6 - 34.9 % (11). Preterm birth accounts for 75% of prenatal deaths, and half the cases of long-term neurological disorders, as well as exorbitant costs of diagnosis, treatment, and care. It is also a huge challenge in terms of time, energy, expenses, and equipment for families and healthcare staff (8, 12). The consequences of preterm birth can be severe, not just by causing neonatal mortality, but also, by leading to medical and developmental disorders (13). Serious complications of preterm birth include respiratory distress syndrome, necrotizing enterocolitis, intraventicular hemorrhage, patent ductusarteriosus, sepsis, and severe and long-term risk of disabilities including mental retardation, epilepsy, blindness, deafness, cerebral palsy, learning disabilities and emotional problems and death(3, 14-19).A wide range of reasons and socio-demographic factors have been reported to affect preterm labor (19). Yet these risk factors cannot properly predict who experiences preterm delivery(20). The high rates of preterm births in poor societies indicate that the reasons must be sought amongst biological and psychosocial factors. In today’s world, non-medical determinants of health are attended to. Each of these determinants by themselves or through affecting one another, strongly influence health status, leading to injustice in health status (21). According to the World Health Organization’s conceptual framework of social factors affecting health, psychosocial conditions like stressors, relationships, and conditions of stressful life, anxiety, depression, and social support are factors that affect health (22). Yet, there is little information available on the effects of psychopathologic factors on consequences of pregnancy (23). Stress is not assessed during routine prenatal care. As a result, the psychological stress level during pregnancy is unclear, and its effect on mother’s health has not been estimated, either (24).Stress, anxiety, and depression are associated with but not just limited to many diseases including cardiovascular, autoimmune, and skin diseases (25). A growing collection of data indicates the adverse effects of psychosocial factors like stress on the consequences of pregnancy (26).Some studies have found that women with higher levels of psychological stress during pregnancy are more exposed to risk of preterm labor or delivering low birth weight infants. Stress is also related to increased susceptibility to infection, nausea, increased blood pressure, and also to undesirable health behaviors such as smoking and alcohol consumption, as mediating factors associated with pregnancy outcomes (27). According to the framework of the World Health Organization, social support is an intermediate factor that can affect mental status of people (22). Perceived social support is individual’s perception of the love and support they receive from family members, friends and acquaintances. Researchers believed that perceived social support is different from received social support (28).Nowadays, this perceived social support has intrigued researchers. Miller believes perceived social support is a better predicting factor for health (29). Previous studies indicate a reduction in mortality rate of people with higher quality and quantity of social network (30). There are a lot of studies on the direct and indirect role (buffer) of perceived social support on reducing stress and improving mental status of individuals. It is believed that social support can directly increase self-esteem, boost resistance against infections, and help behave in a healthy manner. It can also indirectly cause social adjustment and balance individual’s response to stressors and reduce stress, which in turn causes physical and mental health (22, 31-33).Published articles in recent years reveal controversial findings, which have brought few guidelines for physicians, and as for preterm labor risk factors, they do not offer any credible scientific framework. Consequently, recent studies have utilized existing medical records to examine the role of health behavior, history, and individual characteristics of preterm labor(12). They have either solely concentrated on biomedical factors such as complications of pregnancy and exposure to drugs, so the effects of psychosocial factors have not been investigated in the model, or instead of being the main component, they have been considered as confounding factors. Also, little attention has been paid to interaction of these factors with one another in terms of effects on preterm labor (34).

## 2. Objectives

The current study aimed to examine the perceived social support trend in pregnant mothers, and also to analyze stress, anxiety, depression, and other psychological factors with preterm labor.

## 3. Materials and Methods

A prospective longitudinal cohort study was carried out on 550 pregnant women from June 2012 to February 2013. Tehran city was divided into 4 geographical zones of North, South, East, and West (the first stage was cluster sampling), and a public hospital was selected from each cluster. By a simple random method, from the women who referred to the clinic, 550 women in their 24th to 32nd gestational week were identified. The aims of the study were explained to them and if they met the inclusion criteria, an informed consent was obtained from the pregnant women and their spouses. Then the initial interview was conducted, and the delivery date was calculated using the first day of the last menstrual cycle (LMP) stated by the mother, or by the ultrasound report in the first half of pregnancy (if LMP was unknown). The questionnaires were completed between 24th and 32nd week of pregnancy, and samples were followed up until delivery time, and pregnancy was investigated in two groups [1.term delivery (after 37 weeks of gestation), 2.preterm delivery (before 37 weeks of gestation)]. Sample size was measured as 3 to 10 samples per variable by having reviewed relevant literature, considering the prevalence of 10% for preterm labor, and taking into account variables of the study (35). The tool used in the study was a questionnaire that included socio-economic factors, psychological factors (stress, anxiety, and depression), and perceived social support, and stressful Life events. The questionnaires were completed by a specially trained team.

### 3.1. Participants

**Figure 1.**
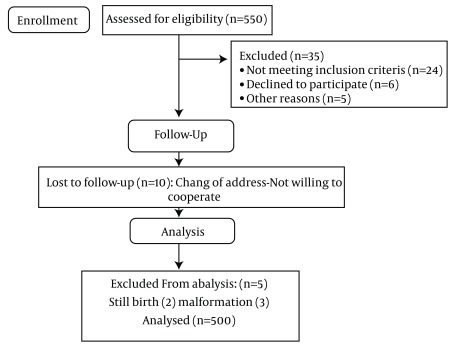
Theoretical Path Model for Effects of Psychological and Socio-Economic Predictors on Gestational Age

Study inclusion criteria were women with gestational age between 24-32 weeks, with singleton pregnancy, without history of known medical problems, both during and before pregnancy such as cardiovascular diseases, diabetes, kidney disorders, respiratory disorders, and autoimmune diseases, and also in a previous pregnancy, problems such as pre-eclampsia, diabetes, premature delivery, intrauterine growth retardation (IUGR), fetal death, premature rupture of fetal membranes (PROM), placental abruption, or polyhydroamniosis. Study exclusion criteria were incidence of pre-eclampsia, diabetes, IUGR, fetal death, PROM, placental abruption, and polyhydroamniosis in the current pregnancy (Figure 1). 

### 3.2. Questionnaire Measures

#### 3.2.1. Demographics and Obstetrics Questionnaire

Mother’s age (based on date of birth), marital status, place of residence (town, village), number of pregnancies and deliveries (gravid/para), number of abortions and still births, planned pregnancy (in view of the pregnant women and husband), history of problems in previous and current pregnancy and Other information such as pre-pregnancy body mass index (BMI) and Hb and HCT (mg/dl) was extracted from the obstetrical records of the pregnant women.To assess the socio-economic status, a questionnaire was designed including the education level of the pregnant mother, the education level of the spouse, area of their place of residence per number of people in a household, cost per square meter of their house, facilities and leisure (having a private car and computer). In this questionnaire, the correlation of these parameters with total score was found to be 0.87. Using factor analysis and summary index, the total standardized score for all subjects was calculated, and using Kappa test, its compliance with normal summary index was investigated. Therefore, the potential maximum score in summarized index was 48 marks(36).The psychosocial status of participants was determined through studying five different dimensions: perceived social support, depression, anxiety, stress, and stressful life events.

#### 3.2.2. Perceived Social Support Questionnaire

Multidimensional Scale of Perceived Social Support (MSPSS)(37) is a 12-item scale that measures perceived social support in three domains of family, friends, and significant others. Participants were asked to indicate their agreement with items on a 7-point Likert-type scale, ranging from very strongly disagree to very strongly agree. Scores range from 12-84, and scores from 12-48 indicate low social support, scores 49-68 indicate moderate social support, and scores 69-84 indicate high social support. Canty-Mitchell and Zimet (2000)(38) evaluated the readability of MSPSS items and found that they were consistent with readings for the fourth grade. Several studies have provided adequate psychometric properties for the MSPSS in young adults and adults in the United States and in Europe (37-39)Canty-Mitchell and Zimet (2000) found the internal reliability of 0.93 for the total score and 0.91, 0.89, and 0.91 for the family, friends, and significant others subscales on a sample of urban adolescents in the United States (40).in Iran Sararoudi (2011) reported Cronbach's alpha0.84 for the scale and 0.90, 0.93 and 0.85, respectively for friends, significant others and family subscale (41). In the present study, Cronbach's alpha coefficient and the intra-class correlation coefficient (ICC) for this tool were 0.89 and 0.92, respectively.

#### 3.2.3. Standard DASS-21 Questionnaire: (Depression, Anxiety, and Stress Scale)

This includes 21 items, with 7 items for each domain of depression, anxiety, and stress. The questionnaire was designed in likert style containing options of never, little, medium, and high. The lowest mark for each question is zero and the highest 3. In each section relevant to depression, anxiety, and stress, score of 1-7 indicates a mild level; 8-14 indicates medium level, and 15-21 severe level. This questionnaire was first introduced by Lavyband in 1995, and was compared with Beck’s Depression Inventory in a large sample and showed a high correlation (r=0.74). Crawford compared this tool with two others and reported its reliability in the domain of depression 0.95, in the anxiety domain 0.91, and in the stress domain 0.93, with overall score of 0.97. This questionnaire has been used in various studies and in different countries and its reliability and validity were examined and confirmed in Iran (42-45).

#### 3.2.4. Stressful Life Event Questionnaire

**Figure 2.**
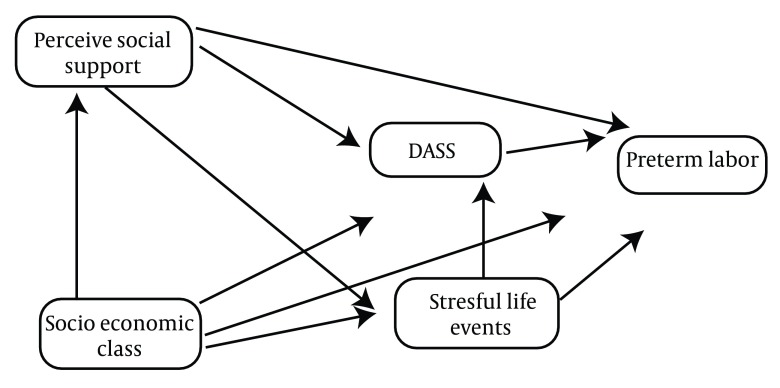
Consort Flow Diagram

This scale was designed by Thomas Holms and Richard Rahe in 1963, and consists of 41 stressful situation items, and determines people’s stress scores by measuring their life changes in the past year. The overall score less than 150 indicates low stress and a good mental health, 150 to 200 indicates moderate level of stress, and 200 to 300 indicates high stress level, and more than 300 indicates sever stress level ( 46 - 48 ).In the current study a conceptual model (Figure 2) was designed to determine the simultaneous relationship between parameters; socioeconomic status, perceived social support, stress and anxiety and depression and stressful life events in pregnancy with incidence of preterm labor. 

Path analysis method was used to examine model good fit and also percentage coverage of variance. Path analysis is a generalization of regression method that is capable of expressing direct, indirect, and total effects of each variable on the dependent variable, and rationally describes the relationships and correlations observed among them. In relation to fitness indices of models in path analysis, the chi-square to degree of freedom index (χ2 / df) less than 3 is preferred, even though some consider values of 4 and even 5 to indicate a good fit. Other indices for fitting the model include: Normed Fit Index (NFI), Comparative Fit Index (CFI), and Goodness Fit Index (GFI), with preferred values over 0.9 (35).In the Root Mean Square Error of Approximation (RMSEA) criteria, values equal to or less than 0.05 indicate a good fit, and values up to 0.08 are acceptable. Although some sources consider values up to 0.11 acceptable (49).The Standardized Root Mean Square Residual (SRMSR) index with values less than 0.08 indicate acceptable fit(50). The SPSS-16 and Lisrel-8.8 software were used for data analysis with the application of path analysis.

## 4. Results

The mean age of the participating women was 27.46 ± 4.97 years and that of their husbands was 31.73±5.32 years. Ninety percent of them were housewives, and 99.1% lived with their husbands. Nearly 90% (87.9%) of the women and 81.8% of their husbands had high school diploma or less, and 77.5% had an income lower than 8,000,000 Rials, and 57.8% did not own their living place. Almost 55% (55.2%) of the participants experienced their first pregnancy. The mean age of the participants at the time of marriage was 17.29±4.37 years. The incidence of preterm labor was 15.5%.The mean ± SD, maximum and minimum of the tools used in the study are shown in (Table1). 

**Table 1.** Instrument Descriptive of Perceived Social Support, Depression, Anxiety, Stress ,Total Dass-21 and Stressful Life Events

Variable	Mean ± SD	Minimum	Maximum
**Perceive social support**	60.41 ± 13.13	16	84.00
**Depression**	4.51 ± 4.03	.00	19.00
**Anxiety**	5.95 ± 4.31	.00	21.00
**Stress**	7.07 ± 4.23	.00	21.00
**Total DASS 21**	17.54 ± 11.38	.00	58.00
**Stressful life events**	101.98 ± 95.28	.00	603

Bivariate analysis was used in order to measure the correlation of the variables before performing pathway analysis (Table 2). 

**Table 2.** Correlations among preterm labor with Social Support, Depression, Anxiety, Stress, Stressful life events

	Stressful life events	Depression	Anxiety	Stress	Dass-21	Perceive social support	Socioeconomic
**Preterm labor**	.136^a^	-.210^a^	-.210^a^	-.132^a^	.203^a^	.054	-.055
**Socioeconomic**	1	-.213^a^	-.219^a^	-.155^a^	-.216^a^	.250^a^	.133^a^
**Depression**		1	.712^a^	.729^a^	.896^a^	-.349^a^	.139^a^
**Anxiety**			1	.736^a^	.906^a^	-.276^a^	.155^a^
**Stress**				1	.910^a^	-.269^a^	.303^a^
**Dass-21**					1	-.328^a^	.221^a^
**Perceive social support**						1	-.091
**Stressful life events**							1

^a^Correlation is significant at the 0.01 level

As indicated in Table 2, gestational age had an inverse significant correlation with socioeconomic status, depression, anxiety, stress, and total DASS-21. Furthermore, depression, anxiety and stress had an inverse significant relationship with social support of the pregnant mother. The model fitness indices obtained in the Table 3 show that the study conceptual model has a good fit. The hypothesis related to causal relationships or the effectiveness of mother’s mental state and also social support on infant’s age at birth have been confirmed. Considering that the model’s mean square errors (0.06) is less than 0.8, and that ratio of chi-square to degree of freedom (2.7) is also less than 3, therefore the model has high fitness and compatibility, which is indicating that the adjusted relationships of variables are based on the theory(Table 3). 

**Table 3.** Goodness of Fit Indices for the Model

Model index	χ2	Df	RMSE	GFI A	NFI	CFI	IFI
	8.41	3	0.064	0.99	0.95	0.96	0.96

**Figure 3.**
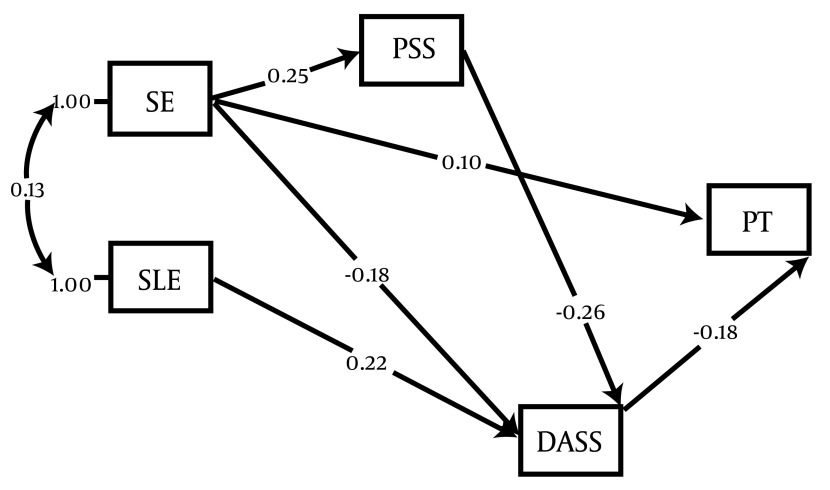
Full Empirical Model (Empirical Path Model for Effects of Stressful Life Events, Socioeconomic Status, Perceived Social Support, Dass-21on Preterm Labor

The effect of variables socioeconomic status, depression, anxiety, stress (DASS-21), perceived social support, and stressful life events on gestational age was studied in pathway analysis (Figure 3). 

As indicated in the figure, DASS-21 among the direct pathways (β = -0.18), and stressful life events among the indirect pathways (_Β_v = -0.0396) had the highest effect on gestational age. Social support directly through socioeconomic status (β = 0.25) and indirectly through stress, depression and anxiety (β = -0.26) affected the gestational age at birth (β= 0.0468). Individual’s socioeconomic status affected gestational ages both directly (β = 0.1) and indirectly through social support, stress, depression and anxiety (β = 0.0441) (Table 4). 

**Table 4.** Path Coefficients for Psychosocial Variable on Gestational Age

Predictor variables	Effects			Model coefficients	T value
	Direct	Indirect	Total		
**Socioeconomic**	0.1	0.0441	0.1441	0.050	2.05^a^
**Dass-21**	-0.18	-	-0.18	0.045	S^a^
**Perceive social support**	-	0.0468	0.0468	0.23	5.92^a^
**Stressful life events**	-	-0.0396	-.396	0.026	5.10^a^

^a^Significant at the 0.05 level

## 5. Discussion

Pathway analysis showed that psychological factors including stress, depression and anxiety had the most direct effects on preterm labor. In addition, stressful life events during pregnancy indirectly affected this pathway. Most studies in this regard, confirm the positive direct effect of psychological stresses such as death of the spouse or close relatives, or divorce on pregnant women, and their indirect effect on unfavorable pregnancy outcome (19, 51-53).The present study showed that perceived social support did not directly affect gestational age. Pathway analysis showed that socioeconomic status directly affected social support. That means, if socioeconomic status is better, the person will enjoy better social support, and consequently has less depression, anxiety and stress. By reducing psychological stressors, the length of pregnancy increases. Studies have shown a significant relationship between social support and health. People who enjoy higher social support have better health status, too (54).Researchers believe that physiologic reactions to stress change under the influence of social support, that is, people’s reaction is less severe among friends and relatives as compared with the time when they face stress alone (55). Two processes have been studied in order to determine the effect of social support on health. The first process expresses the direct effect of social support on health. Accordingly, the positive effects of support or lack of support caused by social isolation directly affect people’s health. The second process operates through moderating effect. Accordingly, social support has no direct effect on health, but moderates the effects of acute and chronic stress on people’s health. It has been known for a long time that experiencing stressors like accidents endangers some people’s health (not all people). It is assumed that the causative effect of life events on causing diseases is moderated through supportive factors like social support. Although most probably the vulnerability factors such as lack of support predispose people to acquire diseases after a stressing event, there is a lot of evidence for both direct effect and moderating effect (56). Psychological stress, affects pregnancy outcome in two different behavioral and physiologic mechanisms. The behavioral mechanism covers lifestyle and unhealthy behaviors like smoking, drug use and immobility. The physiologic mechanism is related to women’s high stress, and women’s and fetal neurohormonal system (57). When someone is faced with stressors like need and/or threat, two physiologic cascades are ignited. One is related to autonomic nervous system and catecholamine release especially norepinephrine and epinephrine. The other one is related to hypothalamus-hypophysis-adrenal axis, which predominantly causes release of corticotropin-releasing hormone, adrenocorticotropin, and cortisol (58, 59). The response of the fetal hypothalamus-hypophysis axis causes an increase in cortisol level, which increases neuromuscular responses and oxytocin release. This increase leads to early uterine muscular contractions and subsequent adverse consequences like preterm labor (53, 60). The activation of both autonomous systems causes both physiologic responses and behavioral changes like reduced appetite and food consumption, decreased sexual activity, increased anxiety and depression, increased insomnia, and agitation (61). In addition, laboratory studies have shown that pregnant women experience a higher level of proinflammatory cytokines like IL-6 and TNF-α if they report higher levels of stress as compared with those who did not. Since IL-10 causes maintaining pregnancy and producing progesterone, it plays an important role in normal pregnancy. These changes are potentially important because increased levels of proinflammatory cytokines and reduced levels of anti-inflammatory cytokines are associated with preterm labor and pre-eclampsia. These findings point to the possibility that stress can increase pregnancy complications indirectly by predisposing immune system to infection or directly by producing proinflammatory cytokines (21, 62). It seems that psychological factors like depression, stress and anxiety directly affect preterm labor, and social support lengthens pregnancy through its moderating effect and reducing the imposition of psychological factors. The present study found a direct relationship between socioeconomic status and social support, mental status and preterm labor. The social causation theory mentions that people’s social class and lifestyle cause mental diseases. In other words, although all people face mental stress, the amount of this stress is different in different social classes. Furthermore, all people do not have access to the same resources in order to cope with stress. Therefore, being in a lower social class can cause conditions that make the individual vulnerable to mental disorders (22). Given the results, it seems that high socio-economic position and an increase in perceived social supports can reduce mental disorders as an intermediate factor, which boosts health status and consequently reduces preterm labor.
